# Neurocognitive impairment, employment, and social status in radiotherapy-treated adult survivors of childhood brain tumors

**DOI:** 10.1093/nop/npab004

**Published:** 2021-01-22

**Authors:** Tiina M Remes, Emma Hovén, Niina Ritari, Heli Pohjasniemi, Riina Puosi, Pekka M Arikoski, Mikko O Arola, Päivi M Lähteenmäki, Tuula R I Lönnqvist, Marja K Ojaniemi, V Pekka Riikonen, Kirsti H Sirkiä, Satu Winqvist, Heikki M J Rantala, Marika Harila, Arja H Harila-Saari

**Affiliations:** 1 Department of Pediatrics and Adolescence, PEDEGO Research Unit and Medical Research Center, Oulu University Hospital and University of Oulu, Oulu, Finland; 2 Department of Child Neurology, Children’s Hospital, University of Helsinki and Helsinki University Hospital, Helsinki, Finland; 3 Department of Women’s and Children’s Health, Karolinska Institute, Stockholm, Sweden; 4 Department of Pediatrics and Adolescence, Kuopio University Hospital, University of Eastern Finland, Kuopio, Finland; 5 Department of Pediatrics, Tampere University Hospital and University of Tampere, Tampere, Finland; 6 Department of Pediatrics and Adolescent Medicine, Turku University Hospital, and Turku University, Turku, Finland; 7 Department of Pediatrics and Adolescence, Helsinki University, and Helsinki University Hospital, Helsinki, Finland; 8 Department of Neurology, Oulu University Hospital, Oulu, Finland; 9 Department of Women’s and Children’s Health, Uppsala University, Uppsala, Sweden

**Keywords:** brain tumor, employment, neurocognitive impairment, radiation, social status

## Abstract

**Background:**

Little is known of the cognitive functions, employment, and social status in adult survivors of childhood brain tumor (BT). We aimed to determine the long-term neurocognitive profile of radiotherapy-treated adult survivors of childhood BT and the relationship between cognitive functions and employment and social status.

**Methods:**

Neurocognitive profiles of survivors were assessed in a Finnish national cohort of 71 radiotherapy-treated survivors of childhood BT (median follow-up time: 21 years [range: 5-33 years]) using a cross-sectional design. Neurocognitive outcomes were compared to control (n = 45) and normative values. Tumor- and treatment-related data were collected from the patient files. Information on employment and social status was gathered.

**Results:**

Survivors’ (median age: 27 years [range: 16-43 years]) median verbal and performance intelligence quotient (IQ) was 90 (range: 49-121) and 87 (range: 43-119), respectively. The cognitive domains with the greatest impairment were executive functions (median *z* score, −3.5 SD [range: −25.0 to 1.3 SD]), and processing speed and attention (median *z* score, −2.5 SD [range: −24.9 to 0.5 SD]). Executive functions were associated with employment, educational level, living independently, having an intimate relationship, and having a driving license. Processing speed and attention were related to educational level, living independently, having an intimate relationship, and having a driving license. Performance IQ was associated with educational level and employment status. Working memory was associated with educational level and living independently.

**Conclusions:**

Radiotherapy-treated adult survivors of childhood BT experience significant neurocognitive impairment, which is associated with difficulties related to employment and social status.

Cognitive impairment is a well-recognized late effect of childhood brain tumor (BT).^1–4^ The BT itself and its location have an impact on the neuropsychological outcome, but treatment and other moderators could also play an important role.^2,4–7^ As the number of adult survivors of childhood BT continues to grow, understanding the long-term neuropsychological effects and their impact on employment and social status is important.

The full-scale intelligence quotient (FSIQ) has shown to be affected after childhood BT and its treatment.^1,2,4–6,8,9^ Both performance and verbal intelligence quotients (PIQ and VIQ, respectively) have been lower among survivors of childhood BT than in the general population, with greater impairment in PIQ than in VIQ.^1,2,4,5,9^ The most impaired domains of the neurocognitive profile were attention, speed, executive functions, memory, and motor dexterity.^10,11^

Cranial radiotherapy was the strongest predictor of poor cognitive outcome in survivors of childhood BT.^4^ A decline of 1-4 FSIQ points per year has been detected following radiotherapy,^1,2,5,9,12^ which has been shown to be a consequence of the inability to acquire new skills and information at the same rate as their healthy peers.^9^ A study of 20 survivors of medulloblastoma showed stable IQ scores after 20-40 years of follow-up, but their working memory continued to decline.^11^

The ability to function in everyday life continues to be affected years after treatment for childhood BT, with reports of a lower educational level and employment rate in survivors than in the general population.^13–16^ These difficulties with employment and social status have been recognized to be related to cognitive issues, but few studies have examined the link between neurocognitive skills, employment, and social status.^13,15–17^ Further, fewer survivors of childhood BT, especially after radiotherapy, get married compared to the survivors of other childhood cancer types; survivors also have poorer peer relationships compared to their siblings.^15,17,18^ Parents have reported that childhood BT survivors experience impairment in social skills related to cooperation, assertiveness, and responsibility, and executive function impairment in survivors has been related to the compromised social skills.^19^

Studies on neurocognitive outcome in long-term adult survivors of childhood BT are scarce, and most have focused on survivors of medulloblastoma.^11,20^ In this study, we investigated the neurocognitive performance in a Finnish national cohort of young adult survivors of radiotherapy-treated childhood BT in a cross-sectional setting and assessed the relationship between their neuropsychological skills and employment and social status. The hypothesis of the present study was that VIQ, PIQ, and executive functions were related to employment and social statuses of the survivors. The findings of this study will enhance the current understanding of the neuropsychological and social functioning of long-term adult survivors of childhood BT, which may lead to future improvements in school-based supportive services and daily living for survivors.

## Materials and Methods

### Participants

The national cohort of consecutive survivors of childhood BT who were diagnosed between 1970 and 2008 and treated with radiotherapy were identified from the registers at the Oulu, Kuopio, Turku, Tampere, and Helsinki University hospitals, where all childhood BT are treated in Finland. The inclusion criteria were the following: (i) BT was diagnosed at ≤16 years of age, (ii) cranial radiotherapy was part of the treatment, (iii) age at the time of the study ≥16 years, (iv) follow-up time since cessation of all tumor therapies ≥5 years, and (iv) no other progressive BT known at the time of the study. All other treatments, for example, operation, shunt operation, and chemotherapy, were allowed. Our work was a part of a more extensive study concerning late complications in survivors of childhood BT in Finland that was conducted from 2010 to 2015.

### Healthy Controls

A total of 45 individuals were randomly assigned from the local population registry of individuals without a history of cancer for our earlier study.^21^ Neuropsychological examination was performed by neuropsychologists (M.H. and S.W.).

### Data Collection

Survivors underwent a 2-day clinical visit during which neuropsychological and clinical examinations were conducted. Treatment-related data were retrieved from the patient files. Educational level, employment situation, marital status, driving license, and living situation were gathered by a questionnaire during the follow-up visit.

### Neuropsychological Examination of Neurocognitive Domains

For intellectual functions, the Wechsler Adult Intelligence Scale (WAIS)-III was used to measure the VIQ and PIQ by the seven subtests.^22^ The similarities, arithmetic, digit span, and information subtests were used to measure VIQ.^22^ The picture completion, coding, and block design subtests were used to measure PIQ.^22^

Processing speed and attention and executive functions were measured by the Trail Making A and B tests, respectively.^23^ Participants were instructed to complete both Trail Making tests as accurately and quickly as possible; then, the completion time was measured.^23^ In case of error, the examiner asked the participant to return to the circle where the error occurred and continue.^23^

For memory functions, the Wechsler Memory Scale-III (WMS-III) was used to measure memory functions.^24^ The Logical Memory I and the Verbal Paired Associates I subtests were used to calculate the Immediate Auditory Memory Index, and the Logical Memory II and the Verbal Paired Associates II subtests were used to calculate the Delayed Auditory Memory Index.^24^ The WAIS-III digit span backward subtest was used to measure working memory.^22^ Visual memory was measured by the Benton Visual Retention Test, modified by Vilkki.^25^ The visuospatial construction was studied using the Rey-Osterrieth complex figure copy test.^26,27^

### Analysis

We performed statistical analysis using the *z* scores of the neuropsychological subtests. We calculated the *z* scores from the standard scores by using the defined means and the SDs provided in the test batteries. In the Wechsler Intelligence Scale, the IQs have values of 100 ± 15, and in all subtests, the values were 10 ± 3.^22,24^ Both in the immediate auditory and delayed auditory memory, the provided means and SDs were 20 ± 6.^24^ Finnish norms for WAIS-III and WMS-III were used in the analysis.

In the absence of the test norms in the Trail Making tests, we used the means and SDs of the normative Canadian population.^28^ For the Trail Making A test, we used the values of 22.93 ± 6.87, 24.40 ± 8.71, and 28.54 ± 10.09 in the age groups of 18-24, 25-34, and 35-44 years, respectively.^28^ For the Trail Making B test, the respective means and SDs were 48.97 ± 12.69, 50.68 ± 12.36, and 58.46 ± 16.41 in the age groups of 18-24, 25-34, and 35-44 years, respectively.^28^ In the absence of the normative values in two 16-year-old participants, we used the normative data of the 18- to 24-year age group.^28^ In the absence of normative values, we calculated the *z* scores for the Benton Visual Retention test by using the mean value and SD of the controls. An American study for normative values for the Rey-Osterrieth Complex figure copy test (32.83 ± 3.10) was used to calculate the *z* scores.^27^ In the absence of normative values in survivors aged <30 years, we used values of 32.83 ± 3.10.^27^

The patients were categorized according to the American Academy of Clinical Neuropsychology consensus statement into low average (*z* scores −1.341 to −0.706 SD) or higher score (*z* score > −0.706 SD), below-average score (*z* scores −2.054 to −1.405 SD), and an exceptionally low score (*z* score < −2.054 SD) groups.^29^

The chi-square exact test (χ ^2^) was used to compare the distributions of categorical data. Due to the non-normal distribution of data, continuous data were analyzed using the Mann-Whitney *U* and Kruskal-Wallis tests and Spearman’s rank correlation. When comparing means of the test results with population norms (means and SDs), we used the Student’s *t* test. To identify factors that predict significantly below-average or exceptionally low score in the neuropsychological tests, significant relationships in the bivariate analyses were further examined using binary logistic regression. For all outcomes, the dependent variable was classified according to the *z* score classifications, where impairment was indicated by a below-average or exceptionally low score (*z* score ≤ −1.405 SDs).^29^ A *P* value of <.05 was used to denote statistical significance.

Statistical analyses were performed using IBM SPSS Statistics for Windows, version 25 and 26 (IBM Corp., Armonk, NY, USA). We excluded two survivors from the analyses concerning employment and social status due to young age (<18 years) at the time of data collection.

### Ethics

Written informed consent was obtained from all participants or their legal guardians. The study was approved by the institutional review boards of the Oulu, Kuopio, Turku, Tampere, and Helsinki University Hospitals, Finland. The research was conducted according to the principles of the Declaration of Helsinki.

## Results

### Patient Characteristics

A total of 71 (56%) participants of the 127 initially eligible survivors of radiotherapy-treated childhood BT, attended the psychological evaluation. Three subjects with consent were not able to participate in the neuropsychological examination due to vision (n = 2) and cognitive (n = 1) impairments.

A total of 40 eligible survivors declined to participate and 13 were lost to follow-up. Data related to patient characteristics, tumors, and treatments did not differ between participants and nonparticipants (Table 1).

**Table 1.** Patient, Tumor, and Treatment Characteristics in All Participants and Nonparticipants

	Participants	Nonparticipants	*P*
Number of participants	71	56	
Age at diagnosis in years			
Median	8.4	8.4	.624^a^
Range	1.1-15.7	0.1-15.7	
Age at follow-up visit in years			
Median	27.2	28.4	.444^a^
Range	16.2-43.8	17.8-49.7	
Follow-up time in years			
Median	20.7	21.8	.210^a^
Range	5.0-33.1	6.6-45.1	
Sex, n (%)			
Male	46 (65)	28 (50)	.106^b^
Female	25 (35)	28 (50)	
Histology, n (%)			
Glial cell tumor	26 (37)	15 (27)	.449^b^
Embryonal tumor	23 (32)	19 (34)	
Ependymoma	8 (11)	8 (14)	
Germ cell tumor	6 (9)	5 (9)	
Tumor of sellar region	3 (4)	0 (0)	
Other	2 (3)	3 (5)	
No histology	3 (4)	6 (11)	
Total dose of radiotherapy			
Median	52.8	53.2	.390^a^
Range	30.0-65.4	16.0-72.0	
Chemotherapy, n (%)	45 (63)	38 (68)	.572^b^
Ventriculoperitoneal shunt, n (%)	44 (62)	34 (62)	1.000^b^
Intimate relation, n (%)	25 (35)		
Living independently, n (%)	44 (62)		
Driving license, n (%)	41 (58)		
Education degree, n (%)			
Primary school^c^	20 (29)		
Secondary school^d^	43 (61)		
Higher degree^e^	7 (10)		
Employment	n (%)		
Unemployed or retired	24 (34)		
Student	19 (27)		
Employed	28 (39)		

^a^Mann-Whitney *U* test.

^b^Chi-square exact test.

^c^Nine years of obligatory schooling.

^d^Studies in high school or career school.

^e^University degree.

The median age at diagnosis was 8.4 years (range: 1.1-15.7 years), and at the follow-up visit 27.2 years (range: 16.2-43.8 years). The median follow-up duration from the end of the radiotherapy to the follow-up visit was 20.7 years (range: 5.0-33.1 years). The baseline characteristics are shown in Table 1. Survivors treated with whole-brain radiotherapy had shorter follow-up time than that of survivors treated with local radiotherapy (median follow-up time: 17.2 years [range: 5.0-29.2 years] vs 21.2 years [range: 8.2-33.1 years]; *P* = .006).

### Characteristics of Healthy Controls

A total of 45 healthy controls (20 males and 25 females) had undergone neuropsychological examinations for our earlier study.^21^ Median age at assessment was 22 years (range: 16-42 years). Healthy controls were significantly younger at the time of assessment compared to the survivors (*P* = .010). Both survivor and control groups did not differ significantly in terms of sex (*P* = .335).

### Neurocognitive Profile

**Figure 1.**
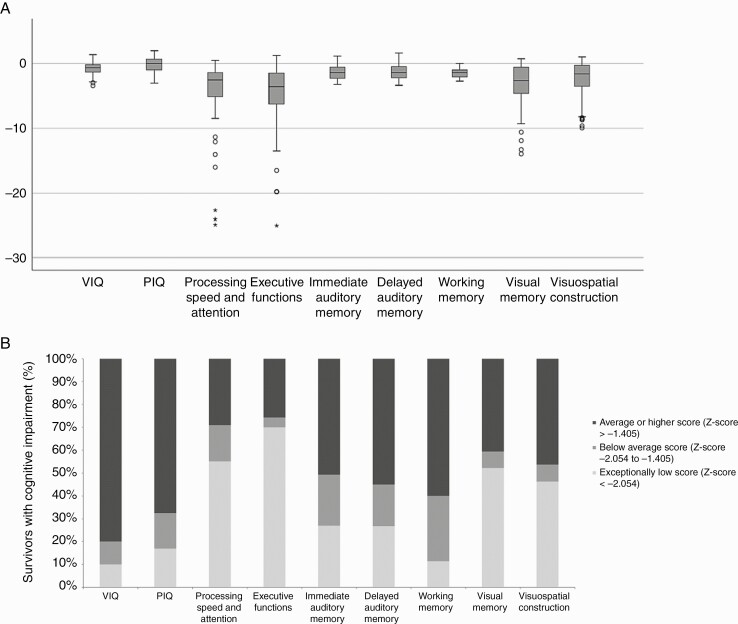
Neurocognitive outcomes in survivors. Neurocognitive outcomes in survivors shown by (A) box plots of *z* scores the lower and upper boundary of the box indicates the 25th and 75th percentiles, respectively; a line within the box marks the median, whisker bars represent lowest and highest values, and outlying and extreme values are shown as circles and crosses, respectively; (B) proportion of survivors showing impaired neurocognitive function, categorized according to the American Academy of Clinical Neuropsychology consensus statement.^29^ Abbreviations: PIQ, performance intelligent quotient; VIQ, verbal intelligent quotient.

The survivors of childhood BT had significantly worse performance in all neurocognitive functions compared to the controls (Table 2). Similarly, when the means of the results were compared to the corresponding population norms, survivors showed lower scores for all neurocognitive functions, except for the picture completion subtest (Supplementary Table S1). Their median VIQ and PIQ scores were 90 (range: 49-121) and 87 (range: 43-119), respectively, while the corresponding scores in the controls were 111 (range: 80-127) and 120 (range: 95-150), respectively. Figure 1 shows the neurocognitive outcome of the survivors in *z* scores and the proportions of neurocognitive impairment. Table 2 presents the standard and *z* scores of the test results. A total of 57 survivors completed all the tests. Only three (5%) of them had a low average score or higher in all neurocognitive domains. Two survivors without neurocognitive impairment were treated with local radiotherapy for infratentorial glial cell tumor (n = 1) and supratentorial ependymoma (n = 1), and one with whole-brain radiotherapy for medulloblastoma. Four (7%) survivors had a below-average or exceptionally low score in one domain: three in processing speed and attention, and one in visuospatial construction. Two or three domains and four domains were scored below average or exceptionally low in 11 (19%) and eight (14%) survivors, respectively. In total, 22 (39%) survivors’ scores were below average or exceptionally low in five to eight domains and for four (7%) survivors in all nine domains.

**Table 2.** Neuropsychological Profile of Childhood Brain Tumor Survivors (CBT) and Healthy Controls (HC)

	Standard Points		*z* Scores		*P*
	CBT (n = 71)	HC (n = 45)	CBT (n = 71)	HC (n = 45)	
	Median (Range)	Median (Range)	Median (Range)	Median (Range)	
VIQ	90 (49-121)^a^	111 (80-127)	−0.7 (−3.4 to 1.4)^a^	0.7 (−1.3 to 1.8)	<.001*
Similarities	9 (1-17)^b^	13 (10-17)	−0.3 (−3.0 to 2.3)^b^	1.0 (0.0-2.3)	<.001*
Arithmetic	9 (2-15)^c^	13 (10-17)	−0.3 (−2.7 to 1.7)^c^	1.0 (0.0-2.3)	<.001*
Digit span	9 (2-16)^c^	13 (7-15)	−0.3 (−2.7 to 2.0)^c^	1.0 (−1.0 to 1.7)	<.001*
Information	9 (1-15)^b^	13 (10-17)	−0.3 (−3.0 to 1.7)^b^	1.0 (0.0-2.3)	<.001*
PIQ	87 (43-119)	120 (95-150)	−0.9 (−3.8 to 1.3)	1.3 (−0.3 to 3.3)	<.001*
Picture completion	10 (1-16)^a^	12 (9-17)	0.0 (−3.0 to 2.0)^a^	0.7 (−0.3 to 2.3)	<.001*
Coding	8 (0-14)^c^	13 (6-18)	−0.7 (−3.3 to 1.3)^c^	1.0 (−1.3 to 2.7)	<.001*
Block design	9 (1-15)^c^	13 (7-19)	−0.3 (−3.0 to 1.7)^c^	1.0 (−1.0 to 3.0)	<.001*
Processing speed and attention^¶^	46 (20-234)^c^	27 (20-39)	−2.5 (−24.9 to 0.5)^c^	−0.6 (−2.2 to 0.5)	<.001*
Executive functions^¶^	95 (38-360)^a^	61 (28-127)	−3.5 (−25.0 to 1.3)^a^	−1.0 (−4.2 to 1.7)	<.001*
Memory functions					
Auditory memory immediate	12 (1-27)^d^	NT	−1.3 (−3.2 to 1.2)^d^	NT	
Delayed	12 (0-30)^f^	NT	−1.3 (−3.3 to 1.7)^e^	NT	
Working memory	6 (2-10)^a^	NT	−1.3 (−2.7 to 0.0)^a^	NT	
Visual memory	20 (3-25)^c^	24 (20-26)	−2.6 (−13.9 to 0.7)^c^	0.1 (−2.6 to 1.4)	<.001*
Visuospatial construction	28 (2-36)	NT	−1.6 (−10.0 to 1.0)	NT	
Age at the study	27 (16-43)	22 (16-42)			<.05*
Sex, n (%)					
Male	46 (65)	25 (56)			.335**
Female	25 (35)	20 (44)			

Abbreviations: NT, not tested; PIQ, performance intelligent quotient; VIQ, verbal intelligent quotient.

*Mann-Whitney *U* test was used here to investigate test results in *z* scores; significant level is 0.05; **Chi-square exact test; ^¶^Time is seconds.

^a^n = 70, ^b^n = 68, ^c^n = 69, ^d^n = 67, ^e^n = 60.

The most marked impairment was found in the executive functions (median *z* score −3.5 SD [range: −25.0 to 1.3 SD]), in processing speed and attention (median *z* score −2.5 SD [range: −24.9 to 0.5 SD]), and in visual memory (−2.6 SD [range: −13.9 to 0.7 SD]). The median *z* score in immediate auditory memory was −1.3 SD (range: −3.2 to 1.2), in the delayed auditory memory −1.3 SD (range: −3.3 to 1.7 SD), in working memory −1.3 SD (range: −2.7 to 0.0 SD), and in visuospatial construction −1.6 SD (range: −10.0 to 1.0) (Figure 1 and Table 2).

### Cognitive Impairment, Employment, and Social Status

Primary school (9 years of obligatory schooling), secondary school (studies in high school or career school), and higher education (ie, university degree) were the highest educational level in 28%, 62%, and 10% of the participants, respectively. Higher impairments in PIQ, processing speed and attention, executive functions, working memory, visual memory, and visuospatial construction were associated with lower survivor educational level (*P* < .05, Table 3).

**Table 3.** Association of Neurocognitive Profile *z* Scores and Employment and Social Status

	Highest educational level				
	Primary school (C) (n = 19)	Secondary school (S) (n = 43)	Higher degree (H) (n = 7)	P*	Pairwise comp.
VIQ, median (range)	−1.0 (−3.4 to 0.3)^a^	−0.5 (−2.2 to 0.8)	−0.2 (−1.3 to 1.4)	.023**	NS
PIQ, median (range)	−1.5 (−3.8 to 0.0)	−0.3 (−2.9 to 1.1)	0.0 (−1.5 to 1.3)	<.001**	C < S, C < H
Processing speed and attention, median (range)	−7.0 (−24.9 to 1.0)	−1.7 (−8.0 to 0.5)^b^	−1.7 (−4.7 to 0.3)^c^	<.001**	C < S, C < H
Executive functions, median (range)	−6.3 (−25.0 to 2.2)	−2.6 (−13.5 to 1.3)^b^	−1.1 (−3.7 to 0.9)	<.001**	C < S, C < H
Working memory, median (range)	−2.0 (−2.7 to 0.3)^a^	−1.3 (−2.7 to 0.3)	−1.3 (−2.0 to 0.0)	.031**	C < S
Visual memory, median (range)	−6.6 (−13.9 to 0.73)^a^	−2.3 (−7.9 to 0.7)^b^	−1.9 (−4.6 to 0.6)	.033**	C < S
Visuospatial construction, median (range)	−2.8 (−10.0 to 0.7)	−1.6 (−9.6 to 1.0)^b^	−0.3 (−1.6 to 1.0)	.038**	C < H
	Employment				
	Unemployed or retired (U) (n = 24)	Student (S) (n = 17)	Employed (E) (n = 28)	P*	Pairwise comp.
PIQ, median (range)	−1.4 (−3.8 to 1.1)	−0.6 (−3.1 to 1.3)	−0.1 (−3.7 to 1.1)	.019**	U < E
Executive functions, median (range)	−4.7 (−25.0 to 0.2)^d^	−3.1 (−19.8 to 0.9)	−1.6 (−19.8 to 1.3)	.015**	U < E
Immediate auditive memory, median (range)	−2.1 (−3.2 to 0.0)	−1.0 (−2.5 to 1.2)^e^	−1.5 (−3.0 to 1.2)^f^	.025**	U < S
Delayed auditive memory, median (range)	−2.0 (−3.3 to 0.3)^g^	−0.5 (−2.8 to 1.7)^h^	−1.2 (−2.7 to 1.2)^d^	.011**	U < S
Visual memory, median (range)	−1.7 (−2.7 to 0.3)	−1.3 (−11.9 to 0.7)	−1.9 (−13.3 to 0.1)	.046**	NS
Visuospatial construction, median (range)	−2.8 (−10.0 to 0.4)^e^	−0.9 (−7.0 to 1.0)^i^	−2.2 (−8.7 to 1.0)^e^	.021**	U < S
Intimate relation	Yes (n = 25)	No (n = 43)		P***	
Processing speed and attention, median (range)	−1.6 (−5.1 to 0.5)^j^	−2.9 (−24.9 to 0.5)		.011**	
Executive functions, median (range)	−2.2 (−25.0 to 0.9)	−4.4 (−19.8 to 1.3)		.048**	
Living independently	Yes (n = 44)	No (n = 25)		P***	
PIQ, median (range)	−0.2 (−3.1 to 1.3)	−1.3 (−3.8 to 0.3)		<.001**	
Processing speed and attention, median (range)	−1.7 (−14.0 to 0.5)^b^	−4.8 (−24.9 to 0.1)		.004**	
Executive functions, median (range)	−2.5 (−10.8 to 1.3)^k^	−5.8 (−25.0 to 0.9)		.003**	
Working memory, median (range)	−1.3 (−2.7 to 0.0)	−1.7 (−2.7 to 0.3)^j^		.018**	
Driving license	Yes (n = 41)	No (n = 21)		P***	
PIQ, median (range)	−0.2 (−2.5 to 1.3)	−1.3 (−3.8 to 0.7)		.004**	
Processing speed and attention, median (range)	−1.6 (−14.0 to 0.5)	−4.5 (−24.9 to 1.0)^l^		<.001**	
Executive functions, median (range)	−2.5 (−10.8 to 1.3)	−4.4 (−25.0 to 0.8)		.006**	

Abbreviations: NS, not significant; Pairwise comp., pairwise comparison; PIQ, performance intelligence quotient; VIQ, verbal intelligence quotient.

*Kruskal-Wallis test.

**Statistically significant (*P* < .05).

***Mann-Whitney *U* test.

^a^n = 18; ^b^n = 42; ^c^n = 6; ^d^n = 23; ^e^n = 15; ^f^n = 27; ^g^n = 22; ^h^n = 14; ^i^n = 16; ^j^n = 24; ^k^n = 43; ^l^n = 20.

Approximately 41%, 35%, and 24% of the survivors of childhood BT were employed, unemployed or retired, and students, respectively. Survivors’ unemployment rate was 16%. Being retired or unemployed was significantly associated with PIQ, executive function, immediate and delayed auditory memory, and visuospatial construction (Table 3).

Approximately 11%, 14%, and 10% of the survivors were married, lived with their partner, or were dating, respectively. Survivors without a current intimate relationship (65%) had a worse performance in processing speed and attention as well as in executive function (Table 3). Among all survivors, 36% did not live independently, which was associated with poorer performance in PIQ, processing speed and attention, executive functions, and working memory. The survivors of childhood BT without a driving license (36%) had higher impairment in PIQ, processing speed and attention, and executive functions (Table 3). We excluded the survivors of childhood BT who were not able to obtain a driving license due to the young age at the time of assessment (n = 2), uncontrolled epilepsy (n = 6), or visual impairment (n = 1).

### Patient, Tumor, and Tumor Treatment Characteristics

**Figure 2.**
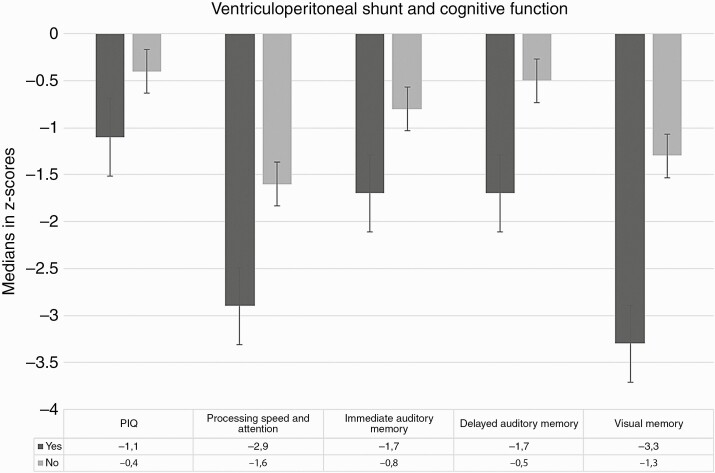
Association between cognitive functions in survivors with and without a ventriculoperitoneal shunt. The Mann-Whitney *U* test was used for analysis; significant associations (*P* < .05) are shown.

**Figure 3.**
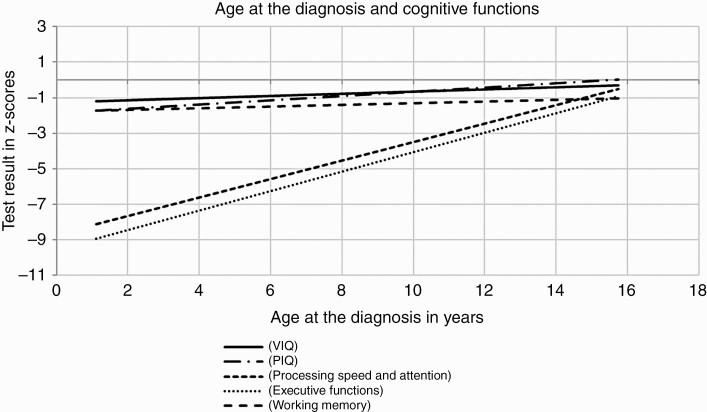
Association between age at the diagnosis and neuropsychological test results. Spearman’s rank correlation was used for analysis; significant correlations (*P* < .05) are shown.

We did not find associations between anticancer treatment modalities and neurocognitive skills. The PIQ performance was worse in participants with infratentorial tumors than in those with supratentorial tumors (Supplementary Table S2). The ventriculoperitoneal shunt was significantly associated with higher impairment in PIQ, processing speed and attention, immediate and delayed auditory memory, and visual memory (Figure 2 and Supplementary Table S2). Age at diagnosis was significantly positively associated with VIQ, PIQ, processing speed and attention, executive functions, and working memory (*P* < .05, Figure 3 and Supplementary Table S3).

The results of the logistic regression analyses showed that a lower age at the diagnosis was significantly associated with impairment in VIQ (odds ratio [OR]: 0.82 [95% confidence interval [CI]: 0.70-0.97]; *P* = .023) and PIQ (OR: 0.81 [95% CI: 0.69-0.97]; *P* = .018). No predictor showed a statistically significant independent association with processing speed and attention. Older age at the follow-up visit was the only factor significantly associated with impairment in executive functions (OR: 0.87 [95% CI: 0.78-0.98]; *P* = .024). The ventriculoperitoneal shunt was associated with impairment in immediate auditory memory (OR: 3.96 [95% CI: 1.39-11.26]; *P* = .010), delayed auditory memory (OR: 4.40 [95% CI: 1.45-13.32]; *P* = .009) and visual memory (OR: 5.49 [95% CI: 1.91-15.85]; *P* = .002). In the multi-variable model for working memory, age at diagnosis was an independent significant predictor (OR: 0.86 [95% CI: 0.75-0.98]; *P* = .025).

## Discussion

We found a high rate of cognitive impairment in young adult survivors of radiotherapy-treated childhood BT. Compared to controls and population norms, survivors of childhood BT showed significant impairment in all neuropsychological domains. The highest impairment was found in executive functions, processing speed and attention, and visual memory. Only three survivors of childhood BT showed a low average score or higher in all neuropsychological domains. Cognitive impairment, especially impairment in executive functions and processing speed and attention, was related to difficulties related to employment and social status.

Survivors achieved a lower educational level and showed a lower employment rate than the Finnish general population.^30,31^ During the study period, nearly a third of the Finnish general population older than 15 years had achieved an education level higher than secondary school; in contrast, only 10% of the survivors had achieved a similar level.^30^ Similarly, while approximately 70% of the working-aged general population in Finland was employed, the rate was nearly half of that in the survivors.^31^ Compared to the unemployment rate of BT survivors in two meta-analyses, survivors in the present study were less likely to be unemployed.^13,17^ De Boer et al. showed that American cancer survivors had a three times higher risk of becoming unemployed compared to European cancer survivors.^13^ Higher childhood cancer survivor rejection rates, lower participation rates for part-time work during their teenage years, and a more discriminatory job market in the United States could be possible reasons for this.^13^ In the present study, survivors who were unemployed or retired performed worse in terms of PIQ, processing speed, and attention than employed survivors. Unemployed or retired survivors had higher impairments in executive functions, immediate and delayed auditory memory, and visuospatial construction than survivors who were students. Moreover, educational level was related to skills in PIQ, processing speed and attention, executive functions, working and visual memory, and visuospatial construction. Survivors with primary school as the highest educational level had greater impairment than those with secondary school or higher education. We believe our results are valid, given that intellectual functioning assessed with intelligence tests is a predictor of academic achievement and vocational success,^4^ but also impairment in other neurocognitive domains are involved.

Furthermore, the observed marriage rate was lower compared to that in the general Finnish population and American childhood BT survivors; however, this could be partly explained by the young age of the survivors.^15,32^ Survivors who performed worse in processing speed and attention and in executive functions were less likely to have an intimate relationship. PIQ, processing speed and attention, and executive functions were all associated with living independently and having a driving license, but those survivors who lived independently had better performance in working memory compared to those who did not live independently. Executive functions and processing speed and attention are considered key cognitive skills, and impairment in these key skills appears related to the ability to learn new skills.^4,20^ This may explain the association between impairment in these skills and lower employment rate and social status in the survivors.

In our study, the survivors had lower mean VIQ and PIQ compared to those of controls, although VIQ and PIQ were average or above in half of the survivors. In our results, IQs were lower than those previously reported in American adult survivors of medulloblastoma.^20^

PIQ was associated with educational level, which is in line with the results of a previous study in adult survivors of medulloblastoma.^33^ Survivors with childhood BT with a second degree or higher education had median IQ scores in the normal range. PIQ was associated with employment status, independent living, and having a driving license. The PIQ tests are dependent on motor functions, visuomotor integration and attention, abstract reasoning, and working memory; such skills that are necessary for employment and while driving a vehicle.^4^

Similar to our results, impairment in executive functions has been recognized in long-term survivors of medulloblastoma.^11^ Executive functions are responsible for behavior control, processing related to goal-directed behavior, and control of complex cognition, especially in nonroutine situations.^34^ Skills of executive functions are needed in essential activities of daily living, especially in complex tasks, and impairment in executive functions has been linked to problems with complicated finances, complex cooking tasks, and remembering events in an elderly population with mild cognitive impairment.^35^ Executive functions were associated with employment and social status in our study (ie, educational level, employment, intimate relationship, living independently or not, and driving license status). However, even those survivors who had fewer difficulties related to employment and social status had low median scores in executive functions. In survivors of childhood BT, poorer performance in executive functions has been associated with difficulties in social skills.^19^

Slow processing speed and attention observed in the survivors of our study were associated with educational level, being in an intimate relationship, living situation, and having a driving license. Regarding executive functions, survivors with difficulties related to employment and social status had slower processing speed and attention, compared to those with better employment and social status. This impairment alters the learning of new skills, especially in academic settings,^36,37^ but may also complicate social situations and independent living.

We found that the majority of survivors had an impaired visuospatial construction, but at a lower rate than previously reported in medulloblastoma survivors.^38^ Visuospatial construction was associated with educational achievement, employment status, and driving license status, all of which could be explained by the fact that performance in visuospatial construction tasks requires good skills in executive functions to establish goals, hold them in active memory, and monitor performance.^39^

Memory impairment was a common finding in survivors while both working and visual memories were associated with educational level and immediate and delayed auditory memories with employment status. Survivors with secondary school education as their highest educational level had a better memory than those having primary school as the highest educational level, and surviving students had a better memory than survivors who were unemployed or retired. In a population of elderly individuals with mild cognitive impairment, an influence of memory impairment was found in everyday life functioning.^35^ We observed an association between the ability to live independently and working memory.

Cognitive functions are dependent on long-distance tracts in the white matter of the brain, which can be disrupted by the tumor itself, intracranial pressure, radiation injury, and the effects of chemotherapeutic agents.^40–42^ Decreased volumes of normal-appearing white matter have been reported in survivors of medulloblastoma, with decreases in volume found being associated with lower FSIQ; this suggests that white matter destruction could partially explain the intellectual deficits in the survivors.^43,44^ White matter injury has also been shown to have a role in deficits of executive function and processing speed in survivors of medulloblastoma and childhood cancer.^20,45^ Moreover, radiation injury in the temporal lobes is related to memory impairment, and an association between such injury and radiation doses to the temporal regions has been shown.^46^ Before and during treatment of childhood BT, survivors are exposed to multiple factors that cause injury to the brain, which can be critical to cognition later in life.

The known risk factors for poor neurocognitive function in survivors of childhood BT include radiotherapy in a dose-dependent manner, whole-brain radiation more neurotoxic than local radiotherapy, chemotherapy, longer time since diagnosis, young age at the time of diagnosis, ventriculoperitoneal shunt, and larger tumor size.^4,8,10,47^ In our study, survivors treated with whole-brain radiotherapy did not have poorer cognitive function than those treated with local radiotherapy, in contrast to a previous report by Grill et al.^8^ Survivors treated with whole-brain radiotherapy were followed up for a shorter time than those treated with local radiotherapy. Newer treatment techniques and improved sparing of healthy tissues in those with a shorter follow-up time may explain the lack of significant differences. However, as in earlier studies on neurocognitive functions, we found that lower age at diagnosis was associated with lower VIQ, PIQ, processing speed and attention, executive functions, and working memory, while in the multivariate analysis, only the association with PIQ remained significant.^1,3,5–7,9^

Ventriculoperitoneal shunt was associated with PIQ, attention and processing speed, and immediate and delayed auditory memory. Our results were in line with those of previous studies on survivors of medulloblastoma with ventriculoperitoneal shunt.^48^

Our study had several limitations, including its cross-sectional design and the relatively small number of healthy controls. Furthermore, the healthy controls were significantly younger than the survivors, although the age difference was considered in the *z* score analysis. The healthy controls in our study had a higher performance in the neurocognitive examination than expected in the normative data, which was in line with previous studies using healthy controls.^49,50^ Another limitation was the lack of control test results for all of the neurocognitive tests used; however, the very low *z* scores of survivors indicated a clear impairment. Study limitations also include the absence of the Finnish norms for Trail Making tests, Benton visual retention, and the Rey-Osterrieth copy test. We also lacked norms for some of the youngest participants in this study.^27–29^ Consequently, the Canadian norms for Trail making and the American norms for the Rey-Osterrieth copy test were used in the present study.^27,28^ These Canadian and American norms are in clinical use in Finland.

In conclusion, our findings confirm that the survivors are at a risk for notable cognitive impairment, especially in executive functions, processing speed and attention, which are related both to a lower employment rate and social status. As executive functions—as well as processing speed and attention—played an important role in the employment and social status of the survivors, the reported IQ scores alone cannot fully describe the survivors’ employment rate and social situation. The extensive impairment in the studied neuropsychological domains and difficulties in employment and social status that were observed in survivors in this study highlight the need for long-term follow-up and supportive services for survivors of childhood BT treated with radiotherapy. Studies on interventions that prevent and rehabilitate these late adverse effects are urgently needed.

## Supplementary Material

npab004_suppl_Supplementary_Table_S1

npab004_suppl_Supplementary_Table_S2

npab004_suppl_Supplementary_Table_S3

## Notes

**Conflict of interest statement.** T.M.R. has received financial support for attending symposia from the Cancer Foundation of Northern Finland; Foundation of Arvo and Lea Ylppö, Finland; the Foundation for Pediatric Research, Finland; and PtcBio, Orion Pharma, and Biogenes. P.M.A. has received financial support for attending symposia from AbbVie and Alexion. M.O.A. has received financial support for attending symposia from Sobi and Pfizer. P.M.L. has received financial support for attending symposia from Shire, Sobi, Bayer, and the Cancer Foundation of Finland. T.R.I.L. has received a speaker honorarium from Actelion Pharmaceuticals and Biomarin. M.K.O. has received financial support for attending symposia from Novo Nordisk and Merck. She has received speaker honoraria from Algol, Pfizer, Merck, and Sandoz. She also received a grant from the Emil Aaltonen Foundation (Finland) and the Cancer Society of Finland. V.P.R. has received financial support for attending symposia from Sobi, Pfizer, and Roche. The other authors report no conflict of interest.

## Notes

**Statement of publication.** This manuscript has been part of the academic thesis of Tiina Remes entitled “Signs of Radiation-Induced Accelerated Ageing in Survivors of Childhood Brain Tumors—The Incidence of Cerebrovascular Disease, Neurocognitive Impairment, Secondary Neoplasms, and Low Bone Mineral Density after 18 Years of Follow-up” published in November 2019 at Oulu University Finland.
