# Supplementary material for: Neurocognitive impairment, employment, and social status in radiotherapy-treated adult survivors of childhood brain tumors
Source: Neurooncol Pract. 2021 Jan 22;8(3):266–77. doi: 10.1093/nop/npab004 (PMC8153831; doi:10.1093/nop/npab004)
Supplement: npab004_suppl_Supplementary_Table_S1 [file npab004_suppl_supplementary_table_s1.docx]

**SUPPLEMENTARY TABLE 1.** Comparison of neuropsychological profile of the survivors with population norms

Mean result of the survivors in Z-scores (SD) Mean difference *P^*^*

(95% Confidence interval)

VIQ (*n* =70) -0.75 (0.93) -0.75 (-0.97 to -0.53) <0.001^**^

Similarities (*n* = 68) -0.50 (1.15) -0.50 (-0.78 to -0.22) 0.001^**^

Arithmetics (*n* = 69) -0.27 (0.95) -0.27 (-0.49 to -0.04) 0.023^**^

Digit Span (*n* = 69) -0.37 (0.95) -0.37 (-0.60 to -0.14) 0.002^**^

PIQ (*n* = 71) -0.87 (1.24) -0.87 (-1.16 to -0.58) <0.001^**^

Picture Completion (*n* = 70) -0.25 (1.28) -0.25 (-0.56 to 0.05) 0.104

Coding (*n* = 69) -0.90 (1.10) -0.90 (-1.16 to -0.63) <0.001^**^

Block Design (*n* = 69) -0.52 (1.11) -0.52 (-0.78 to -0.25) <0.001^**^

Processing speed and attention (*n* = 69) -4.31 (5.40) -4.31 (-5.61 to -3.01) <0.001^**^

Executive functions (*n* = 70) -4.98 (5.31) -4.98 (-6.25 to -3.71) <0.001^**^

Immediate auditory memory (*n* = 67) -1.37 (1.10) -1.37 (-1.64 to -1.10) <0.001^**^

Delayed auditory memory (*n* = 60) -1.21 (1.11) -1.21 (-1.50 to -0.92) <0.001^**^

Working memory (*n* = 70) -1.40 (0.65) -1.40 (-1.55 to -1.24) <0.001^**^

Visual memory (*n* = 69) - 3.32 (3.38) -3.32 (-4.14 to -2.51) <0.001^**^

Visuospatial construction (*n* = 67) -2.50 (2.95) -2.50 (-3.22 to -1.78) <0.001^**^

Abbreviations: SD, standard deviation; VIQ verbal intelligent quotient; PIQ, performance intelligent quotient

^*^Comparison of mean result in Z-scores with population norms using Student’s T test

^**^Significant level is 0.05
