# Supplementary material for: Neurocognitive impairment, employment, and social status in radiotherapy-treated adult survivors of childhood brain tumors
Source: Neurooncol Pract. 2021 Jan 22;8(3):266–77. doi: 10.1093/nop/npab004 (PMC8153831; doi:10.1093/nop/npab004)
Supplement: npab004_suppl_Supplementary_Table_S2 [file npab004_suppl_supplementary_table_s2.docx]

**SUPPLEMENTARY TABLE S2.** Association of Neuropsychological Profile and Patient and Tumor Characteristics and Tumor Treatment

Verbal IQ Performance Processing Executive Immediate General Working Visual Visuo-

IQ Speed and Functions Auditory Auditory Memory Memory spatial

Attention Memory Memory Memory Construction

(*n* = 70) (*n* = 71) (*n* = 69) (*n* = 70) (*n* = 67) (*n* = 60) (*n* = 70) (*n* = 69) (*n* = 67)

Tumor Location

Infratentorial (*n* = 38)

Median -0.6 -1.3 -3.4^h^ -4.3 -1.3^h^ -1.5^i^ -1.3 -2.9 -2.2^h^

Range (-3.4 – 1.4) (-3.8 – 1.1) (-24.1 – 0.5) (-25.0 – 0.5) (-3.2 – 1.2) (-3.3 – 1.7) (-2.7– 0.0) (-13.9 – 0.7) (-10.0 – 1.0)

Supratentorial (*n* = 33)

Median -0.7^j^ -0.2 -1.6^j^ -2.8^j^ -1.3^k^ -1.0^l^ -1.3^j^ -1.9^m^ -0.9^k^

Range (-2.2 – 0.8) (-3.7 – 1.3) (-24.9 – 0.5) (-19.8 – 1.3) (-3.0 – 0.7) (-3.0 – 0.7) (-2.7 to -0.3) (-11.9 – 0.7) (-8.5 – 1.0)

*p** .604 .001^**^ .063 .102 .353 .277 .249 .184 .069

Radiotherapy

Local (*n* = 39)

Median -0.5^n^ -0.4 -2.5^o^ -3.2^n^ -1.3^p^ -1.3^j^ -1.3^n^ -1.9^o^ -0.9^q^

Range (-2.8 – 1.4) (-3.8 – 1.3) (-24.9 – 0.3) (-19.8 – 0.9) (-3.0 – 1.2) (-3.0 – 1.7) (-2.7 – 0.0) (-13.9 – 0.7) (-9.6 – 1.0)

Whole-brain (*n* = 32)

Median -0.9 -1.1 -2.5 -4.5 -1.7^m^ -1.3^r^ -1.3 -2.6 -2.7

Range (-3.4 – 0.4) (-3.4 – 1.0) (-22.7 – 0.5) (-25.0 – 1.3) (-3.2 – 1.2) (-3.3 – 1.2) (-2.7 to -0.7) (-13.3 – 0.7) (-10.0 – 1.0)

*p** .111 .170 .427 .128 .236 .381 .101 .502 .074

Chemotherapy

Yes (*n* = 45)

Median -0.9^b^ -0.9 -2.8^b^ -4.3 -1.6^s^ -1.2^o^ -1.3^b^ -2.6^b^ -1.6^t^

Range (-3.4 – 0.6) (-3.8 – 1.3) (-24.9 – 0.5) (-25.0 – 1.3) (-3.2 – 1.2) (-3.3 – 1.7) (-2.7 to -0.3) (-13.9 – 0.7) (-10.0 – 1.0)

No (*n* = 26)

Median -0.4 -0.4 -1.6^u^ -2.9^u^ -1.3^u^ -1.3^e^ -1.3 -2.6^u^ -1.9^d^

Range (-2.6 – 1.4) (-3.1 – 1.1) (-8.5 – 0.3) (-10.5 – 0.3) (-3.0 – 0.7) (-3.0 – 0.3) (-2.7 – 0.0) (-8.6 – 0.7) (-9.6 – 1.0)

*p** .088 .515 .069 .083 .912 .527 .125 .935 .864

Ventriculoperitoneal Shunt

Yes (*n* = 44)

Median -0.7^t^ -1.1 -2.9^t^ -4.0 -1.7^v^ -1.7^i^ -1.3^t^ -3.3^t^ -1.6^s^

Range (-3.4 – 1.4) (-3.8 – 1.3) (-24.9 – 0.5) (-25.0 – 1.3) (-3.2 – 1.2) (-3.3 – 1.7) (-2.7 – 0.0) (-13.9 – 0.7) (-10.0 – 1.0)

No (*n* = 27)

Median -0.6 -0.4 -1.6^w^ -2.6^w^ -0.8 -0.5 -1.3 -1.3^w^ -0.9^u^

Range (-2.2 – 0.8) (-2.9 – 1.1) (-8.0 – 0.3) (-13.5 – 0.9) (-3.0 – 1.2) (-2.8 – 1.2) (-2.0 to -0.3) (-6.6 – 0.7) (-8.3 – 1.0)

*p** .777 .045^**^ .025^**^ .132 .002^**^ .010^**^ .460 <.001^**^ .123

^a^ *n* = 45; ^b^ *n* = 44; ^c^ *n* = 39; ^d^ *n* = 24; ^e^ *n* = 23, ^f^ *n* = 21; ^g^ *n* = 22; ^h^ *n* =37; ^i^ *n* = 33; ^j^ *n* =32; ^k^ *n* = 30; ^l^ *n* = 27; ^m^ *n* = 31; ^n^ *n* = 38; ^o^ *n* = 37; ^p^ *n* = 36; ^q^ *n* = 35; ^r^ *n* = 28; ^s^ *n* = 42; ^t^ *n* =43; ^u^ *n* = 25; ^v^ *n* = 40; ^w^ *n* = 26

* Mann-Whitney U test, ** Significant level is 0.05
