# Supplementary material for: Neurocognitive impairment, employment, and social status in radiotherapy-treated adult survivors of childhood brain tumors
Source: Neurooncol Pract. 2021 Jan 22;8(3):266–77. doi: 10.1093/nop/npab004 (PMC8153831; doi:10.1093/nop/npab004)
Supplement: npab004_suppl_Supplementary_Table_S3 [file npab004_suppl_supplementary_table_s3.docx]

**SUPPLEMENTARY TABLE S3**. Association between neuropsychological outcome in Z-scores and age at the diagnosis, age at the follow-up visit, and follow-up time in all participants and follow-up time in whole-brain radiotherapy treated

Correlation Coefficient *P**

Age at the diagnosis

VIQ (*n* = 70) 0.253 0.035**

PIQ (*n* = 71) 0.395 0.001**

Processing speed and attention 0.468 <.001**

(*n* = 69)

Executive functions (*n* = 70) 0.447 <.001**

Immediate auditory memory (*n* = 67) 0.066 0.596

Delayed auditory memory (*n* = 60) -0.093 0.480

Working memory (*n* = 69) 0.280 0.019**

Visual memory (*n* = 69) 0.172 0.158

Visuospatial construction (*n* = 67) 0.110 0.377

Follow-up time

VIQ (*n* = 70) -0.080 0.511

PIQ (*n* = 71) 0.018 0.879

Processing speed and attention 0.066 0.589

(*n* = 69)

Executive functions (*n* = 70) 0.091 0.456

Immediate auditory memory (*n* = 67) -0.118 0.341

Delayed auditory memory (*n* = 60) -0.151 0.250

Visual memory (*n* = 69) -0.187 0.125

Visuospatial construction (*n* = 67) 0.029 0.818

*Spearmann rank-order correlation analysis was used. ** Significant level is 0.05

Abbreviations: VIQ = Verbal intelligent quotient; PIQ = Performance intelligent quotient
